# Methods for Determining the High Molecular Weight of Hyaluronic Acid: A Review

**DOI:** 10.3390/polym17243289

**Published:** 2025-12-11

**Authors:** Amanda Esperanza López-Cánovas, Miguel Victoria-Sanes, Ginés Benito Martínez-Hernández, Antonio López-Gómez

**Affiliations:** 1 Institute of Plant Biotechnology, Universidad Politécnica de Cartagena, Campus Muralla del Mar, Edificio I+D+I, 30202 Cartagena, Spain; amanda.lopez@upct.es (A.E.L.-C.); miguel.victoria@upct.es (M.V.-S.); 2 Food Safety and Refrigeration Engineering Group, Department of Agricultural Engineering, Universidad Politécnica de Cartagena, Paseo Alfonso XIII, 48, 30203 Cartagena, Spain

**Keywords:** viscosity, agarose electrophoresis, size-exclusion chromatography, polydispersity

## Abstract

Hyaluronic acid (HA) is a key extracellular biopolymer whose molecular weight (MW) critically determines its physicochemical behaviour and biological functionality. Accurate MW characterization is therefore essential for applications ranging from tissue engineering to medical and cosmetic formulations. Although previous reviews have addressed general aspects of HA, they have only briefly summarized analytical approaches for MW determination. In contrast, the present review provides the first dedicated and comparative analysis of the three most widely used and practically relevant techniques—agarose gel electrophoresis, size-exclusion chromatography coupled with HPLC (SEC-HPLC) and intrinsic viscosity measurements. We examine recent methodological advances, including improved calibration strategies, optimized gel matrices, multi-detector SEC configurations, and updated empirical models for viscometric analysis. Particular emphasis is placed on how these innovations enhance accuracy, reproducibility and applicability across different HA size ranges and sample purities. By critically evaluating the strengths, limitations and sources of analytical bias of each technique, this review offers a consolidated framework to guide researchers and industry professionals in selecting the most appropriate method for reliable MW assessment. Overall, we highlight how methodological refinement is enabling more robust characterization of HA, even in highly polydisperse or complex samples.

## 1. Introduction

The terms hyaluronic acid and hyaluronan are often used interchangeably. Technically, hyaluronan refers to the anionic salt form present under physiological conditions, whereas hyaluronic acid is the protonated form. However, “hyaluronic acid” remains the more commonly used term in both scientific literature and commercial contexts. Hereinafter, we will refer to hyaluronic acid as HA.

In 2024, the global HA market was valued at 9.76 billion € and is projected to reach 15.20 billion € by 2030, registering a compound annual growth rate (CAGR) of 7.81% from 2025 to 2030. This growth is driven by the increasing demand for anti-ageing treatments and the rising awareness of HA’s benefits for skin hydration, wound healing, and joint health. North America led the market with a 42.1% share in 2024, while the Asia Pacific region is expected to be the fastest-growing over the forecast period. In particular, the dermal fillers segment stands out as the leading application, accounting for 41.8% of total revenue in 2024 [1].

HA is a linear non-sulfated glycosaminoglycan that plays a fundamental role in the extracellular matrix of vertebrate tissues. HA is a high-molecular-mass polysaccharide composed of repeating D-glucuronic acid and N-acetyl-D-glucosamine units linked by alternating β-1,4 and β-1,3 glycosidic bonds, reaching molecular weight sizes up to 10 MDa [2]. Indeed, it is of high interest to use accurate molecular weight techniques, being the most relevant (as discussed later), agarose gel electrophoresis, size-exclusion chromatography coupled with high-performance liquid chromatography (SEC-HPLC) and intrinsic viscosity. The molecular weight of HA determines its wide applications in several sectors (medical, cosmetic, pharmacology, food, etc.), like tissue (bone, cartilage, peripheral nervous system, etc.) engineering, wound healing, cancer treatments, food additives, delivery systems, among others, as recently reviewed [3,4].

HA can be produced by several methods, such as microbial production (native or toxin-free genetically modified bacteria), cell-free production (chemical and/or enzymatic methods) or extraction from animal tissues [4,5]. The isolation of produced HA, which involves complex protocols (like those historically employed in DNA purification), is needed due to limitations of short HA chains as well as a wide polydispersity of the final HA product, released toxins during microbial production, etc. [3]. Indeed, the HA price is mainly affected by its purity and molecular weight, which also determine its applications in different sectors [6]. For example, medical applications suitable for injectable applications like osteoarthritis require high-purity HA, which may drive the price up to 30-fold higher (1730 €/kg for topical HA vs. 43,200 €/kg for injectable HA according to the US International Trade Commission and Scientific Literature) [7]. The main factors influencing production costs are the production titer, the recovery yield, and the scale of the bioreactor, with recombinant production needing to at least double its efficiency to compete with production with wild strains [7,8].

Several reviews have covered a wide list of HA aspects, such as structure, sources, physicochemical properties, quantification methods, functionalities/applications, etc. [3,4,5,9,10,11,12,13,14,15]. In particular, Cowman et al. [9], Rivas et al. [11] and Wu et al. [15] included sections in their reviews that provided an overview of certain analytical techniques for determining the molecular weight of HA. Nevertheless, there is still a lack of a dedicated review focusing specifically on the most relevant techniques for HA molecular weight determination. Accordingly, this review examines agarose gel electrophoresis, size-exclusion chromatography coupled with high-performance liquid chromatography (SEC-HPLC) and intrinsic viscosity measurements for determining the molecular weight of HA.

Polydispersity is an inherent feature of hyaluronic acid samples, as they consist of chains with a distribution of molecular weights rather than a single, well-defined size. The degree of polydispersity strongly influences the rheological behaviour, biological activity, and analytical response of HA. Therefore, accurate molecular-weight characterization requires methods capable of capturing this distribution and not only average values, especially in highly polydisperse or partially degraded samples. Although the present review focuses on the techniques themselves, acknowledging the relevance of polydispersity provides context for interpreting their performance and limitations.

### 1.1. Structure and Physicochemical Properties of Hyaluronic Acid

**Figure 1:**
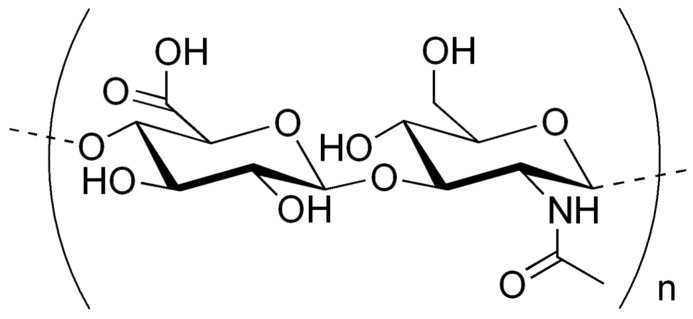
Chemical structure of hyaluronic acid showing its repeating disaccharide units of D-glucuronic acid and N-acetyl-D-glucosamine linked alternately by β-1,4 and β-1,3 glycosidic bonds.

The HA structure is composed of repeating disaccharide units of D-glucuronic acid and N-acetyl-D-glucosamine, linked alternately by β-1,4 and β-1,3 glycosidic bonds [(1 → 3)-β-D-GlcNAc-(1 → 4)-β-D-GlcA] (Figure 1). This configuration results in a high-molecular-mass polysaccharide—represented as ([-3)GlcNAc(β1-4)GlcA(β1-]n)—that can reach molecular sizes up to 10 MDa, making it one of the largest biopolymers found in nature [2].

The specialized structural organization of HA is directly responsible for its distinctive physicochemical and biological properties, which underpin its widespread application in biomedical and therapeutic fields (such as medicine, cosmetics, food, and health care). This unique molecular structure defines the properties that make HA a highly valuable biomaterial, as explained below:

(i) The HA exhibits remarkable viscoelasticity and hydrophilicity, driven by its high density of hydroxyl and carboxyl groups. These structural features allow it to retain substantial quantities of water, which is essential for maintaining tissue hydration and lubrication [15].
(ii) It also shows excellent biocompatibility and non-immunogenicity, primarily due to its ubiquitous presence and conserved structure across human tissues. These characteristics enable HA to be well-tolerated by the body, even when derived from exogenous sources [16,17].
(iii) It is fully biodegradable, mainly through enzymatic cleavage by hyaluronidases and oxidative mechanisms, generating oligosaccharides that may retain biological activity and functional relevance [18].
(iv) Moreover, HA demonstrates native bioactivity, as it interacts with several cell surface receptors—including CD44 and RHAMM—mediating critical physiological processes such as cell adhesion, proliferation, migration, and modulation of inflammatory responses [19].

Together, these features support HA’s broad biological functionality across various tissues and underline its wide-ranging biomedical applications [4].

### 1.2. Molecular Weight of Hyaluronic Acid and Its Biological Implications

Importantly, HA is inherently polydisperse, and it has been demonstrated that the biological activity of HA is closely associated with its molecular weight [4]. The degree of polydispersity, expressed as the ratio *Mw*/*Mn*, can be measured using size-exclusion chromatography (i.e., SEC-HPLC).

In industrial settings, polydispersity is often minimized through fractionation, enzymatic degradation, or ultrafiltration. It is crucial to have a low polydispersity in the sample since the molecular weight determines the HA application. For instance, high-molecular-weight HA is required for viscosupplementation or wound healing [20]. However, in applications such as drug delivery or cancer therapy, low-molecular-weight HA may be more suitable [21]. It must be taken into consideration that, to reduce polydispersity, the production cost increases; thus, the decision to pursue monodisperse HA has to be analyzed in terms of economic feasibility.

#### 1.2.1. High-Molecular-Weight Hyaluronic Acid (HMW-HA)

High-molecular-weight HA (HMW-HA), typically defined as having a molecular weight ≥1 MDa, is predominantly found in healthy tissues (skin, cartilage, synovial fluid, and vitreous humour) as well as in animal-derived materials like rooster comb and rabbit muscle. Generally, HA occurs in the high-molecular-weight range of 2 to 6 MDa [22] and exhibits anti-inflammatory effects. It suppresses cell proliferation and differentiation, downregulates the production of pro-inflammatory cytokines and reduces macrophage-mediated phagocytosis [23,24,25,26]. Structurally, HA can retain significant amounts of water due to its ability to form intramolecular hydrogen bonds, resulting in a three-dimensional network. This contributes to its ability to form hydrogels with excellent lubricating, moisture-retaining, and water-absorbing properties. These characteristics support critical cellular processes such as migration, adhesion, and proliferation [27]. Moreover, the molecular weight of HA significantly influences its functional performance. Higher molecular weight correlates with increased resistance to enzymatic degradation by hyaluronidase, enhanced water-binding capacity, and prolonged residence time in the body [28]. Due to these properties—particularly its viscoelasticity, hydration retention, and lubricating function—HMW-HA is widely applied in clinical and cosmetic settings. In medicine, it serves as a primary component in intra-articular injections to restore joint lubrication and mitigate cartilage degeneration. In aesthetic procedures, it is commonly used as a base for dermal fillers [23,28,29].

The global market of HA has experienced sustained growth over the last decade due to its various applications. The growing interest in anti-ageing products and articular therapies has significantly boosted the global demand for HA. Depending on the molecular weight, the cost per gram of HA can range from tens to hundreds of euros, with HMW-HA being considerably more expensive due to the complexity of its production and purification. The price also depends on its source and purity, which ultimately determines its final application.

#### 1.2.2. Medium- and Low-Molecular-Weight Hyaluronic Acid (MMW-HA and LMW-HA)

Medium-molecular-weight HA (MMW-HA; 1 × 104 Da–1 MDa) contributes to wound healing and embryonic development, whereas low-molecular-weight HA (LMW-HA; <1 × 104 Da) plays a key role in chronic wound healing and the development of HA crosslinker [30]. LMW-HA is predominantly found in biological fluids (e.g., blood, saliva, milk, urine, amniotic fluid) and in tissues exposed to inflammation, ageing, irradiation or neoplastic transformation [9]. Unlike HMW-HA, LMW-HA has the ability to act as a pro-inflammatory marker, promoting the activation and maturation of dendritic cells and the release of pro-inflammatory cytokines such as interleukin-1β and tumor necrosis factor-α [24,26]. Moreover, LMW-HA enhances cell mobility [31]. The use of LMW-HA is more common in the cosmetic field, although it also has important application prospects in food, healthcare and medicine [13].

In addition to these biological functions, the physicochemical properties of MMW-HA and LMW-HA differ markedly from those of HMW-HA, and these differences largely explain their distinct applications. As chain length decreases, the polymer exhibits a reduced hydrodynamic radius, lower solution viscosity and diminished water-binding capacity, which translate into faster diffusion through tissues and lower resistance to enzymatic or oxidative degradation. MMW-HA therefore displays intermediate viscoelastic behaviour and a shorter residence time compared to HMW-HA—characteristics that favour physiological processes such as angiogenesis, fibroblast migration and tissue remodelling during wound healing. Its moderate stability and enhanced tissue penetration also make it suitable for topical formulations and regenerative therapies where controlled and temporary activity is desired.

LMW-HA (<10 kDa), on the other hand, behaves less as a structural polymer and more as a signalling molecule. Its small size facilitates interaction with receptors such as CD44, RHAMM, TLR2 and TLR4, activating pro-inflammatory and immunomodulatory pathways. This molecular form is known to stimulate cytokine release, promote cell motility and modulate immune cell behaviour, which explains its involvement in chronic wound environments, inflammation-driven tissue repair and tumour microenvironment regulation. Due to its high permeability and rapid clearance, LMW-HA is widely incorporated into cosmetic products aimed at enhancing dermal penetration and hydration, and is increasingly used in drug-delivery systems where rapid systemic distribution is advantageous. However, these same properties limit its suitability for applications requiring mechanical support or long-term lubricating functions.

From an analytical standpoint, the lower viscosity and reduced molecular entanglement of MMW-HA and LMW-HA influence their behaviour in electrophoretic and SEC-based methods, typically yielding sharper bands and later elution volumes compared with HMW-HA. Moreover, LMW-HA is particularly susceptible to artefactual chain scission during sample handling, which underscores the need for careful method selection and controlled preparation when characterizing low-molecular-weight fractions.

### 1.3. Biosynthesis of Hyaluronic Acid: Eukaryotic and Microbial Pathways

HA is a linear polymer, and both its chain length and molecular weight are set during its biosynthesis by hyaluronan synthases. These enzymes catalyze the polymerization of the sugar precursors UDP–glucuronic acid and UDP–*n*-acetylglucosamine at the inner side of the plasma membrane. In vertebrates, three isoforms (*HAs1*, *HAs2*, and *HAs3*) are involved. Each of them has different enzymatic activities and produces HA with a different molecular weight, usually ranging from around 100 kDa up to more than 6 MDa [32,33]. In some bacteria, the enzyme *HasA* performs a similar function and can produce HA of comparable size [34].

The final molecular weight distribution of HA highly depends on the producer organism, the culture conditions, and the genetic tools used to improve production. These factors can lead to HA samples with different levels of polydispersity. Given the strict standards required for medical uses, having reliable analytical techniques to measure the molecular weight and quality of HA is crucial [16,35].

**Figure 2:**
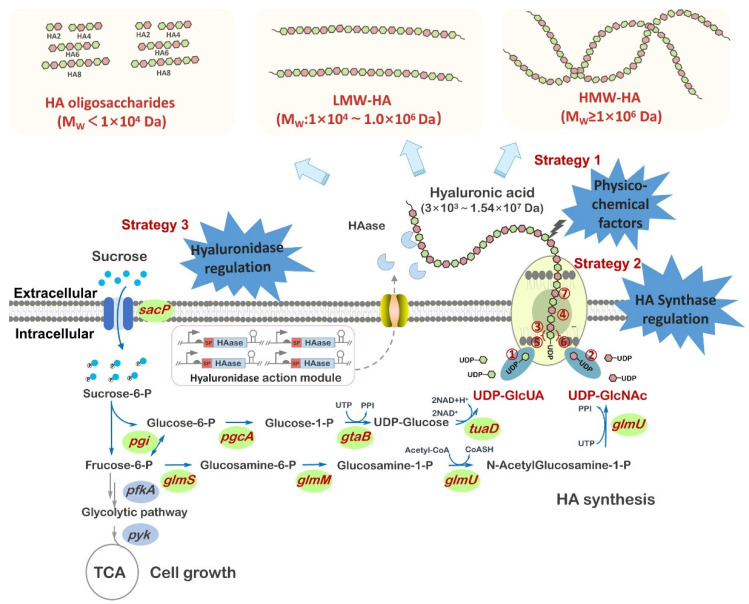
Schematic diagram of the currently available strategies for the regulation of the molecular weight of hyaluronic acid (HA) [37]. The biosynthesis pathway of HA is as follows: sucrose is transported into the cell through the PTS system and then 6-P-glucose and 6-P-fructose are formed by sucrose 6-phosphate hydrolase SacA; UDP–glucuronic acid is synthesised by 6-P-glucose in the presence of phosphoglucose modifase PgcA, UDP–glucose pyrophosphorylase GtaB (HasC), and UDP–glucose dehydrogenase TuaD (HasB); UDP–*N*-acetyl-D-glucosamine is synthesised by transamidase GlmS, transacetylase GlmM, and pyrophosphorylase GlmU; and the UDP–glucuronic acid and UDP–*N*-acetyl-D-aminosaccharide precursors synthesise HA by HA synthases. HAs contain seven structures: ①, ② UDP-GlcUA and UDP-GlcNAc binding sites; ③, ④ the supply sites of HA-GlcUA-UDP and HA-GlcNAc-UDP; ⑤, ⑥ UDP-GlcUA and UDP-GlcNAc glycosyltransferase sites; ⑦ HA transmembrane site.

This high variability of the molecular weight of HA is closely regulated at the biosynthetic level, where the enzymatic machinery determines both the polymer length and, consequently, its physiological role. Thus, understanding the mechanisms that govern HA synthesis and size distribution is essential to fully appreciate its functional and biomedical relevance [19,32]. HA is synthesized at the inner face of the plasma membrane by a class of enzymes known as hyaluronan synthases (*HAs*), as observed in Figure 2. In vertebrates, three *HAs* isoforms are involved: *HAs1*, *HAs2* and *HAs3* [32]. These enzymes use cytoplasmic nucleotide-sugar precursors—UDP–glucuronic acid (UDP-GlcA) and UDP–N-acetylglucosamine (UDP-GlcNAc)—as substrates. These precursors are synthesized through central metabolic pathways: UDP-GlcA from UDP–glucose and UDP-GlcNAc from fructose-6-phosphate via the hexosamine biosynthesis pathway. The synthesis reaction involves the alternating addition of these sugars to the growing HA chain by the *HAs* enzymes, and the polymer is simultaneously extruded into the extracellular space. The overall reaction can be summarized as: *n* UDP-GlcA + *n* UDP-GlcNAc → HA*_n_* + 2*n* UDP. Each HAs isoform differs in its expression levels, the rate of HA synthesis, and the molecular weight of the HA produced: *HAs1* synthesizes high-molecular-weight HA (>2 MDa), but at a low rate, *HAs2* produces very-high-molecular-weight HA (up to 6–8 MDa) and is essential for embryonic development and structural functions. *HAs3* produces HA with lower molecular weights (100 kDa to 1 MDa) but at a higher rate compared to *HAs1* and *HAs2* [36].

### 1.4. Sources of Hyaluronic Acid: Bacterial Production vs. Animal Tissue Extraction

HA extraction from animal sources faces several challenges, including harsh processing conditions that reduce yield and affect molecular uniformity [12,13]. Additionally, animal-derived HA may be contaminated with proteins like hyaluronidase, which can trigger immune responses [36,38], and there is a risk of infectious agents such as nucleic acids, prions, or viruses [36]. The process is also time-consuming, labour-intensive and costly. According to the cost breakdown for HA production, the purification process with 50% represents the most significant expense of the total production cost (fermentation materials, 15%; culture media, 10%; personnel costs, 10%; electricity, 8%; packaging and storage, 7%). This high proportion can be attributed to the complexity of removing impurities such as proteins, nucleic acids, endotoxins, and other by-products that accumulate during fermentation. Purification steps often require multiple unit operations, including centrifugation, filtration, precipitation, and chromatography, which increase operational time, labour and material consumption.

These limitations make microbial production a safer and more efficient alternative. Accordingly, microorganism-based HA production seems to be an ideal approach, presenting low production cost and more effective purification [39].

Recently, demands for HA products from bacterial fermentation have significantly increased because of both their increased uses as medical devices and the immune issues that have occurred from the use of animal-based HA. Due to both the high price of HA and the high standard requirements of its applications in medical products, high-quality HA products rather than high quantity have been the primary criteria used when selecting the bacterial strains used for HA production and the methods of HA purification [16]. Hence, the molecular weight and purity of HA are key indicators of its quality, with polymers exceeding 0.5 MDa possessing greater market value [35].

Certain Gram-positive bacteria, especially *Streptococcus* spp. belonging to Lancefield classification group A or C, naturally produce HA as part of their extracellular capsule. This HA is chemically identical to that in vertebrates and serves as a virulence factor by mimicking host tissues and evading immune detection [40]. In bacteria, HA synthesis is carried out by HAs. This enzyme is encoded by the HAs gene in the has operon, often along with HAsB (UDP–glucose dehydrogenase) and HAsC (UDP–glucose pyrophosphorylase), which help generate the necessary precursors [40,41]. Molecular weight can vary depending on strain, culture conditions, and genetic engineering, but typically, natural strains produce HMW-HA (500 kDa–2 MDa) and engineered strains up to 4–6 MDa [34]. The HA yield could reach 6–7 g/L (litre of fermentation media), approaching the upper technical threshold of the process, primarily limited by mass transfer constraints resulting from the elevated viscosity of the fermentation broth. Although microbial fermentation enables the production of high-purity HA, the process is still susceptible to contamination by bacterial endotoxins, residual proteins, nucleic acids, and trace heavy metals [42].

An alternative that has been more extensively studied is the use of microbial production hosts, particularly those classified as “Generally Recognized As Safe” (GRAS), for industrial-scale HA biosynthesis [35,43,44]. Nevertheless, the molecular weight of HA produced by a non-genetically modified GRAS microorganism is low [5].

Genetic engineering can introduce the operon responsible for HA biosynthesis into relatively safe host strains. The recombinant organisms thereby acquire the capacity to produce HA. Among GRAS-designated strains, *Bacillus subtilis* is most employed; however, *Lactococcus lactis*, *Escherichia coli*, and the widely used food-grade *Streptococcus thermophilus* are also considered viable hosts for HA production. In addition, *Cornybacterium glutamicum* is widely employed for HA production due to its ability to achieve high titers under optimized fermentation conditions [45].

### 1.5. Analytical Relevance of Hyaluronic Acid’s Molecular Weight

The molecular weight of HA significantly influences the choice and performance of analytical techniques used for its determination. Validated analytical methods provide confidence and certainty in the results, are part of good analytical practices, are a requirement of regulatory agencies and pharmacopoeias and save time and resources. However, the advantages and limitations of the available different molecular weight analysis methods for HA must be studied in detail to obtain an accurate molecular weight characterization, depending on the HA sample.

**Intrinsic viscosity** is effective for HMW-HA, as larger molecules contribute more to solution viscosity. The intrinsic viscosity method is approved as the only molecular weight determination method for HA by the European Pharmacopoeia. However, this method becomes less sensitive for LMW-HA, where viscosity changes are minimal.

**Agarose gel electrophoresis** is suitable for separating HA molecules based on size, with a linear relationship between electrophoretic mobility and the logarithm of molecular mass observed for HA in the range of approximately 200 to 6000 kDa [46]. The agarose gel electrophoresis method is also contemplated in the American Pharmacopoeia.

**Size-exclusion chromatography** coupled with high-performance liquid chromatography (**SEC-HPLC**) offers robust quantification and distribution profiling of HA molecular weights, with the ability to analyze a wide range of sizes. However, the accuracy of SEC-HPLC analyses can be influenced by factors such as calibration standards and sample concentration, which may lead to variations in molecular weight estimations [47]. Hence, the molecular weight range of HA plays a crucial role in determining the most appropriate analytical method, as each technique has specific advantages and limitations depending on the size of the HA molecules being analyzed.

## 2. Methods for Analyzing the Molecular Weight of Hyaluronic Acid

The natural form of HA in all organisms is in its polydisperse form. Nevertheless, the HA function is highly influenced by its molecular weight. For example, HMWHA is anti-inflammatory, whereas LMWHA is pro-inflammatory, exhibiting the same dichotomy for other processes such as cell migration/invasion, angiogenesis and joint lubrication, among others (as previously compiled by Nguyen et al. [48]). Hence, a simple, sensitive and accurate method for characterizing the molecular weight of HA has been intensively investigated and remains a challenge. Among them are gel electrophoresis, viscosimetry, size-exclusion chromatography (SEC) coupled to different detectors (refractive index, UV or multiangle laser light scattering (MALLS)), gas-phase electrophoretic mobility molecular analysis (GEMMA), enzyme-linked sorbent assay (SEC-ELSA), Matrix-Assisted Laser Desorption/Ionization–Time of Flight (MALDI-TOF), field flow fractionation (FFF) and nanopore analysis, among others.

In Table 1 are summarised the advantages and limitations of all those techniques for the HA molecular weight determination. Among them, gel electrophoresis is currently the most widely available, inexpensive and easily implemented method to characterise the molecular weight in a wide range of HA sizes (from a few kDa to at least 6 MDa) of imperfectly pure HA specimens [9,10,11]. Contrary, the other techniques (SEC-MALLS, capillary electrophoresis, MALDI-TOF, GEMMA, etc.) require highly purified HA samples. In addition, SEC-MALLS, MALDI-TOF and solid-state nanopore techniques require expensive equipment and highly trained personnel. SEC-HPLC coupled with refractive index and/or UV index detectors have been used to identify the molecular weight within linear ranges of 100–270 kDa to 2–2.5 MDa. This technique uses readily accessible equipment, requires minimal personnel training, is quick and easy to run, and is cost-effective. Finally, viscosimetry (using a Ubbelohde or Canon viscosimeter) remains the most reliable technique for evaluating the molecular weight of HA. Hence, the viscosimetry method has been standardized and published in the European and Japanese Pharmacopoeias. However, viscosimetry only provides an average value of HA molecular weight rather than its distribution.

Therefore, this review focuses on the gel electrophoresis technique and SEC-HPLC to characterize the HA molecular weight distribution, while the standardized and widely accepted viscosimetry method is deeply reviewed as a technique to determine the average value of HA molecular weight.

### 2.1. Viscometry

Measurement of the viscosity of a solution containing HA allows the determination of the polymer viscosity-average molecular weight, which is close to its weight-average [49,50]. Viscosity of a HA solution may be measured either by: (i) viscosimeter (Ubbelohde or Canon viscosimeters), in which the HA solution flows (riven by the gravity force) through a calibrated tube or capillary, and the time elapsed between two marked points is measured; (ii) rheometer, in which the HA solution is placed between a stationary (stator) and a movable (rotor) plate or cylinder, measures the force required to initiate or halt the movement of the rotor element [11]. The viscoelastic properties of HA have been comprehensively reviewed by [49]. Therefore, in this review section, we will focus on the viscometer method, as no recent reviews have deeply addressed the various mathematical models used to estimate the average molecular weight from the time elapsed between viscometer marks.

**Figure 3:**
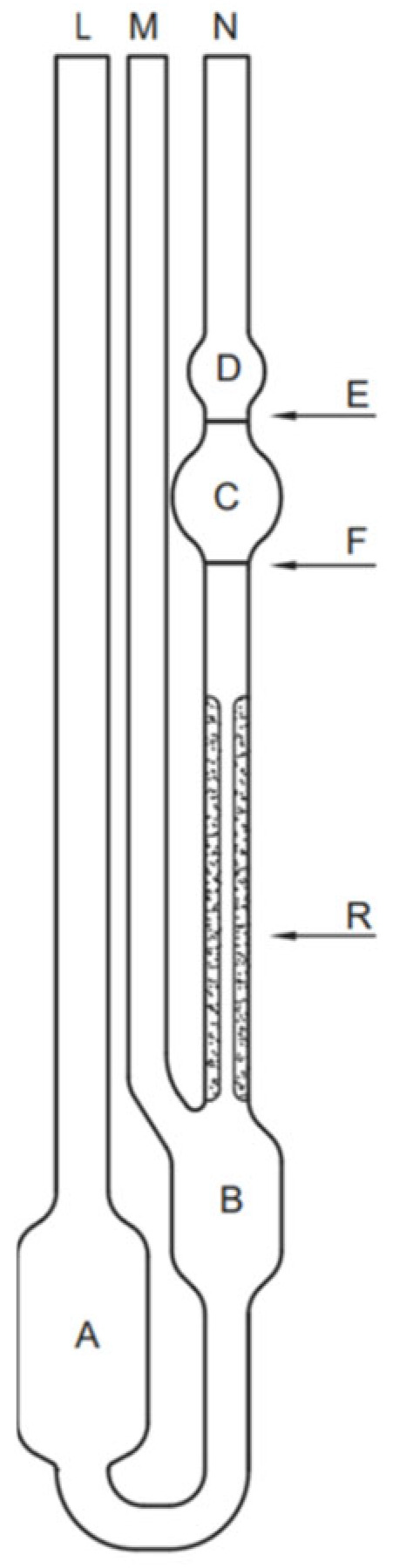
Suspended level Ubbelohde viscometer. The intrinsic viscosity measurement involves recording the time it takes for the test solution (HA in saline) to pass from mark E to mark F and comparing it to the corresponding time for the solvent (saline solution). The sample is introduced through arm L, and a total volume of 15 mL is required to fill bulb B and to reach above the first mark on bulb A. Bulb C corresponds to the lower measuring bulb, and bulb D corresponds to the upper measuring bulb. Mark M indicates a reference level on the side arm, and tube N represents the main tube containing the capillary through which the liquid ascends and subsequently flows downward during the measurement.

For the HA sample preparation, the HA sample should be previously dissolved in physiological salt solution. This is necessary to eliminate contributions to viscosity from intramolecular and intermolecular electrostatic repulsion [49]. Aqueous NaCl and NaNO3 solutions (0.10–0.20 M) are the most frequent solvents used for the molecular weight determination of HA by viscometry [51]. Nevertheless, the differences in the intrinsic viscosity values determined with both solvents are not significant [52]. In addition, the value of intrinsic viscosity depends on the salt concentration of the solvent [53]. In general, NaCl solvent concentrations of 0.15–0.20 M have been studied. Whereas the European Pharmacopoeia (EP) refers to 0.15 M sodium chloride in 0.01 M phosphate-buffered solution, the Japanese Pharmacopoeia (JP) requires 0.20 M sodium chloride solution. After dissolution, the sample should be dialyzed in cold against a large volume of the solvent to establish osmotic equilibrium between the HA solution and the dialysate to be used in making dilutions of the sample. Both the sample and the dialysate may be filtered (membrane with an effective pore size of 0.20–0.45 µm) or alternatively the sample may be centrifuged [50]. These preprocessing steps are optional and are performed only when needed to ensure a homogeneous, particle-free solution for accurate kinematic viscosity measurements. The prepared HA solution is then flowed through a suspended-level viscometer using gravity as the driving force (Figure 3). The experimental setup also includes a constant-temperature bath—specially designed and commercially available for viscometric measurements—capable of maintaining a temperature of 25.0 ± 0.5 °C during the measurements. A suspended-level outflow glass capillary viscometer is used, which allows direct dilution of the sample within the viscometer itself [54]. Regarding the suspended-level viscometer, the European Pharmacopoeia (Ph. Eur. 11.0) recommends the following viscometry specifications: viscometer constant of approximately 0.005 mm^2^/s^2^, kinematic viscosity of 1–5 mm^2^/s, internal diameter of tube 0.53 mm, volume of bulb of 5.6 mL and internal diameter of tube of 2.8–3.2 mm. The viscometer should have a funnel-shaped lower capillary end. It is recommended to use the same viscometer for all measurements.

The force of gravity drives the flow, and the transit time between two calibration marks is measured for both the buffer (*t*_0_) and the test solution (*t*). The test is considered valid only if the results do not deviate by more than 0.35% from the mean value and unless the flow time (*t*) is not less than 1.6 times and not more than 1.8 times t0 (1.6 *t*_0_ ≤ *t* ≤ 1.8 *t*_0_).

The viscosity of the pure solvent and the viscosity of the polymer solution (polymer dissolved in solvent) are set in relation to each other, which leads to relative, specific and reduced viscosity values (Equation Set 1) [55]. In particular, relative viscosity (*η*_rel_) indicates how much more viscous the polymer solution is compared to the pure solvent; specific viscosity (*η*_sp_) indicates how much the viscosity increases due to the presence of the polymer; (*η*_red_) is obtained by dividing *η*_sp_ by the polymer concentration (*C*); and inherent viscosity (*η*_inh_) is an alternative way to calculate viscosity using logarithms.


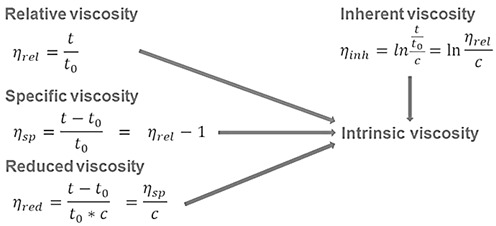


(Equation set 1)

The intrinsic viscosity ([*η*]) is a property of the polymer that measures its contribution to the viscosity of a solution at zero concentration. It is obtained by extrapolating (*η*_red_) and (*η*_inh_) vs. concentration (*C*) as c → 0. The [*η*] can be calculated according to Equation (1):

(1) $$\left[\eta \right]=\frac{\sqrt{\left[\left(\frac{t}{t_{0}}\right)-1-\ln\left(\frac{t}{t_{0}}\right)\right]\times 2}}{C}$$

where *t* and *t*_0_ are the elapsed times of the test and buffer solutions, respectively, and *C* is the HA concentration of the test solution (g/dL). Intrinsic viscosity is also related to the specific volume of the polymer, which, in turn, is associated with the ratio of the hydrodynamic volume of an HA chain to its molecular weight [50,56]. This demonstrates the strong relationship between viscosity, molecular weight and concentration [57]. Since viscosity is a key property of the HA polymer, contributing to its viscoelastic behaviour in the synovial fluid, it has been shown that increasing molecular weight and concentration strengthens HA networks. Consequently, HA solutions display progressively increased viscosities [58,59,60].

Huggins [61] proposed a model for predicting the activity coefficients of polymer solutions, explicitly linking them to the Gibbs free energy [62]. For a well-behaved linear polymer, specific viscosity can be described by the concentration and intrinsic viscosity of the polymer [61]. This model is particularly applicable to systems involving high-molecular-weight polymers [63]. For HMW-HA, (*η*_sp_) is most estimated using the Huggins equation, which relates the specific viscosity-to-concentration ratio (*η_sp_*/*C*) to [*η*] and concentration (*C*) and Huggins constant (*k′)* [64], as observed in Equation (2). The k′ constant has a theoretical value of 0.4 according to the Stokes–Einstein equation for calculating the viscosity of a suspension of spheres [65]. The usual procedure involves plotting *η_sp_*/*C* versus *C*, to obtain a straight line with [η] as the y-intercept and *k*′[*η*]^2^ as the slope.

*η*_sp_/*C* = [*η*] + *k*′[*η*]^2^
*C* (2)

The definition of a sufficiently diluted solution depends on the molecular weight of the polymer. In general, the product [*η*] × C should be <1. If the previous restriction is not met, it may lead to erroneously high *k*′ values and underestimated [*η*] values.

When higher concentrations must be used, [*η*] can instead be estimated by the Martin equation (Equation (3)). In that case, [*η*] is obtained from the intercept of the linear regression line and the decimal antilogarithm of the intercept is the [*η*] expressed in m^3^/kg [66].

ln (*η*_sp_/*C*) = ln [*η*] + *k*′[*η*] *C* (3)

The uncertainty arising from the determination of MW-HA by viscometry can be reduced by using many data points to refine the constants in the Mark–Houwink equation (Equation (4)):

[*η*] = *k* × (M_rm_)*^α^* (4)

It should be noted that the coefficient *k* and the exponent *α* from the Mark–Houwink equation are specific for the polymer molecule and the solvent [67]. This fitting also provides insights into conformational changes in HA as a function of molecular weight [67]. Also, the average molecular mass of HA calculated from the intrinsic viscosity depends on the choice of the Mark–Houwink coefficients [65].

For HMW-HA analyzed in a capillary viscometer with a shear rate of about 1000 1/s, Bothner et al. [68] proposed the use of the Mark-Houwink-Sakurada equation with parameters *k* = 0.397 × 10^−3^ dL/g and *α* = 0.601 (Table 2). Nevertheless, there is an overview of published combinations of *k* and *α* for linear (non-crosslinked) HA dissolved in 0.1–0.2 M aqueous sodium chloride solution [67] (Table 2).

Despite that, the best fit of the rheological characteristics of HA solutions over the entire range of [*η*] can be achieved by the following combination of coefficients (according to private communication of Rolf Bergman (2009) reported by Muller-Lierheim [67]: *k* = 0.13327 × 10^−3^ dL/g; *α* = 0.6691.

Determination of the HA molecular weight by viscometry has several practical challenges, including the requirement for large sample sizes and variability induced by the dependence of viscosity on experimental factors like ionic strength and temperature. Moreover, viscometry can only provide a mean molecular weight of HA rather than its distribution. Still, the industry often uses this technique for product quality control, as it is a simple and well-established method for obtaining average molecular weight information from HA [11].

### 2.2. Agarose Gel Electrophoresis

Agarose gel electrophoresis is one of the most accessible, simple, and cost-effective methods for estimating the molecular weight of HA samples, particularly useful for HMW-HA of up to 6 MDa [11,46,77]. Among the rest of the techniques to determine the HA molecular weight, electrophoresis is one of the most widely adopted for HMW-HA, due to its ease of implementation and minimal equipment requirements [9,64].

Gel electrophoresis provides a practical alternative, leveraging the intrinsic physical and chemical properties of HA for size-based separation. HA molecules possess a constant charge-to-mass ratio due to their uniform repeating disaccharide structure, which includes one negatively charged carboxyl group per disaccharide unit. This characteristic allows HA to migrate through a gel matrix under the influence of an electric field, like DNA or denatured proteins, facilitating size-dependent separation [78]. Although sample purification is important, HA does not require high purity for gel electrophoresis analysis, particularly when specific detection methods such as Stains-All are employed. Nonetheless, samples must be free of strongly bound proteins, which can alter electrophoretic mobility and lead to inaccurate MW estimations [79].

To achieve effective molecular weight resolution, the gel matrix serves as both a sieving medium and a stabilizer of molecular separation. Under appropriate electrophoretic conditions, smaller HA molecules migrate more rapidly, while larger ones experience greater resistance. The range of molecular weights that can be separated depends on the relative size of the HA molecules and the pore size of the gel matrix. The porosity of the gel is primarily determined by the concentration and crosslinking degree of the gel-forming polymer. Gels with small average pore sizes, such as crosslinked polyacrylamide, are suitable for separating LMW-HA oligosaccharides and fragments. Conversely, gels with larger pore sizes—such as agarose gels—are ideal for the resolution of high-molecular-weight HA species [50]. HA standards must be co-electrophoresed alongside unknown samples on the same gel to generate a reliable standard curve for accurate size determination [80].

The separation efficacy depends critically on gel pore size, determined primarily by agarose concentration and buffer composition, which modulate sieving effects. Low-percentage agarose gels (0.5–2.0%) prepared in Tris-borate-EDTA (TBE) or Tris-acetate-EDTA (TAE) buffers are predominantly used to resolve HA within 200 kDa to 6 MDa [46,64,81,82,83,84,85,86,87,88,89]. Agarose gels provide larger pores than polyacrylamide gels, making them preferable for HMW-HA, whereas polyacrylamide gels better resolve LMW-HA [15]. Table 3 compiles the proposed electrophoresis methods and their key analytical parameters.

Recent modifications by Zanjani et al. [81] and Karami et al. [84] used 1% agarose gels in TAE or TBE buffers with varying electrophoretic conditions, including pre-runs and fixation steps before staining with Stains-All, optimizing staining intensity and band resolution. Karami et al. [84] notably used higher Stains-All concentrations (5%) with shorter destaining under light for rapid visualization. Gottschalk et al. [83] revisited Lee and Cowman’s method with minor adjustments, employing 0.5% agarose in TAE at 105 V for 55 min, combined with commercial HA standards and prolonged Stains-All staining, demonstrating reproducible HA MW distributions.

In summary, while all methods utilize the fundamental principles of agarose gel sieving and HA’s charge-to-mass ratio, they differ primarily in agarose concentration (0.5–1.2%), buffer choice (TAE or TBE), electrophoresis duration and voltage (30 V to 105 V; 30 min to 8 h), staining protocols (Stains-All or Toluidine Blue concentrations and times), and destaining procedures. These variables influence resolution, sensitivity, and accuracy of molecular weight determination. Importantly, calibration with appropriate HA standards and controlled staining/destaining are critical for quantitative densitometry. Although newer methods offer improved separation or faster processing, validation against established standards remains essential to confirm accuracy in MW distribution analysis.

### 2.3. Size-Exclusion Chromatography (SEC)

Several configurations of SEC exist (SEC-RID, SEC-UV, SEC-MALS, among others), which are considered variations of the same analytical technique rather than independent methods. For this reason, all these approaches are discussed collectively within the SEC section.

Size-exclusion chromatography (SEC) separates molecules according to differences in their hydrodynamic dimensions, which are determined by both their molecular mass and conformation in solution [90]. Although historically referred to as gel filtration or gel permeation chromatography, the term SEC has become the most prevalent and accurately conveys the basis of the method. The principle was first demonstrated in the mid-20th century with swollen starch matrices and quickly gained traction due to its non-invasive separation mechanism, which preserves molecular integrity and bioactivity, alongside the development of commercially available macroporous media (compiled by Hall [91]). These stationary phases may be fabricated from various materials, including cross-linked natural polymers such as dextran or agarose, synthetic polymers such as polyacrylamide or polymethacrylate, hybrid composites, or silica-based particles. The resolution of an SEC separation depends chiefly on the average pore size and the distribution of pore diameters within the beads, and, ideally, no specific chemical interaction between analyte and matrix occurs. Molecules small enough to penetrate the pores diffuse into them, whereas larger species are excluded. Upon sample application and initiation of elution, the smaller components explore a greater fraction of the accessible pore volume, whereas larger macromolecules traverse a reduced path and elute earlier. Since partitioning is dictated by steric accessibility rather than binding site saturation, the effective loading capacity is related to the sample-to-pore volume ratio, not to the total solute mass. Greater separation efficiency can be achieved by maximizing the fraction of pores within the relevant size range and by increasing the ratio of column volume to applied sample volume [91].

In size-exclusion chromatography coupled to high-performance liquid chromatography (SEC-HPLC), the number-average (${\bar{M}}_{n}$) and weight-average (${\bar{M}}_{w}$) molecular weights are typically used to describe polymer size distribution. The parameter ${\bar{M}}_{n}$ reflects the molar concentration, while ${\bar{M}}_{w}$ is weighted by mass. In a monodisperse polymer, these values are identical; however, the ratio ${\bar{M}}_{n}/{\bar{M}}_{w}$, known as the polydispersity index (PDI), quantifies sample heterogeneity, with higher values indicating broader molecular weight distributions. The general approach involves constructing a calibration curve relating the partition coefficient (*K_av_*) to the logarithm of the molecular weight of standards of known size, typically covering a broad range (e.g., from kilodaltons to several megadaltons). The partition coefficient is defined as described in Equation (5):

(5) $$K_{av}=\frac{V_{e}-V_{0}}{V_{T}-V_{0}}$$

where *V_e_* is the elution volume of the analyte, *V*_0_ is the column void volume, and *V_T_* is the total permeation volume. Calibration data allows estimation of ${\bar{M}}_{n}$ and ${\bar{M}}_{w}$ from chromatographic peak areas corresponding to polymer fractions. From these values, the PDI is calculated, and the degree of polymerization can be derived when the molar mass of the repeating unit is known. This methodology is widely applied to HA and other polysaccharides, following established data analysis procedures to integrate chromatographic peaks, assign molecular weights to fractions, and calculate distribution parameters. The result is a detailed profile of the molecular weight distribution, which is essential for understanding the polymer’s physical properties and functional performance [92].

SEC coupled with HPLC detectors like refractive index (SEC-RID) or absorbance-based (SEC-UV) (considered both as conventional SEC) and SEC coupled with a multi-angle light scattering (MALS) HPLC detector (SEC-MALS), have been the most studied SEC techniques to determine the molecular weight of HA [47,93] (Table 4). Conventional SEC has been considered as a reproducible technique that minimizes the drawbacks associated with light scattering (SEC-MALS), sedimentation equilibrium, and non-Newtonian fluid viscometry of HA solutions, and requires a small sample volume for analysis [48,92]. Hence, Nugyen et al. [48] developed a SEC-UV method sensitive, accurate and reproducible for characterizing (molecular weight) and quantifying HA samples.

#### 2.3.1. Conventional SEC: SEC-RID and SEC-UV

Conventional SEC techniques (RID or UV detectors) have been the preferred methods to determine the molecular weight of HA due to their less expensive nature, tolerance to partially purified HA samples (contrary to SEC-MALS), easy to use, sensitive, accurate and reproducible method [47,48] (Table 4). Two key factors that may limit the accuracy of HA molecular weight determination by conventional SEC techniques are: (i) the calibration standard used and (ii) the injected sample concentration [47,100]. In addition, SEC performance is also influenced by specific HPLC parameters (mobile phase type and concentration, column type, number of columns connected in series, column temperature, detector settings, etc.).

**Figure 4:**
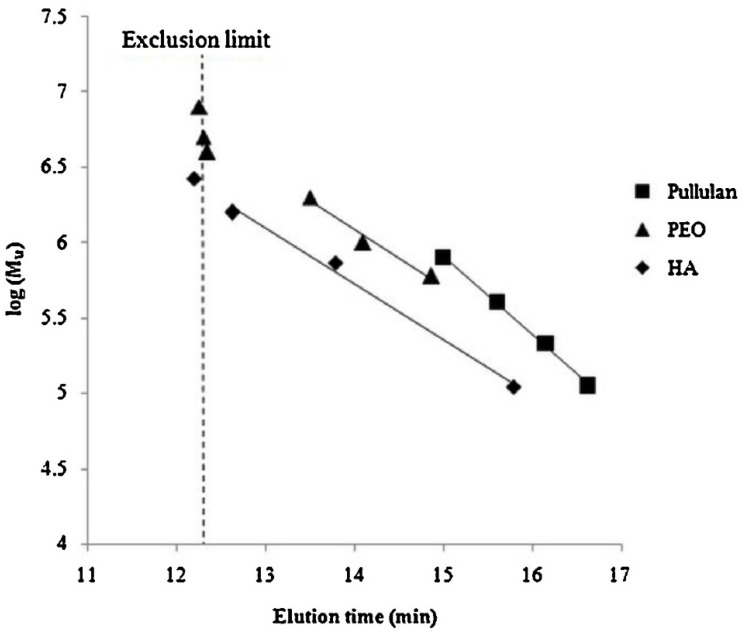
Calibration plot based on HA, PEO, and pullulan molecular weight standards [47].

HA standards for conventional SEC. At present, in contrast to many other biological substances, no certified reference material specific to HA is widely available. In addition, regarding HA standards with low polydispersity, a few HA multi-component standards (known as *ladder* in gel electrophoresis) are commercially available to be used also in SEC-HPLC, covering limited molecular weight ranges (LoLadder, 0.027–0.5 MDa; HiLadder, 0.5–1.5 MDa; MegaLadder, 1.5–6.09 MDa; Select-HA™ products). While organisations such as the NIBSC (National Institute for Biological Standards and Control) supply international standards for numerous biopharmaceuticals, HA remains notably absent from their catalogue. This lack of standardized reference HA standards impedes the direct comparison of SEC analytical data among laboratories and studies, thereby contributing to inconsistencies in quality control practices and regulatory documentation. Due to such scarcity and the high costs of these HA standards with low polydispersity, suboptimal calibration procedures and inaccurate molecular weight estimations are common, such as the use of polydisperse HA standards or universal calibration methods based on non-HA polymers (dextran, pullulan, polystyrene sodium sulfonate, and polyethylene oxide). These frequently used non-HA polymers differ from HA in both chemical composition and molecular conformation, which is only valid for relative or comparative assessments between HA samples [47,101]. Nevertheless, it is common in the literature to treat molecular weight values derived from non-HA calibrants as accurate, and to compare results obtained with different calibration standards, a practice that can lead to significant misinterpretations, as observed in Figure 4 [47].

**Figure 5:**
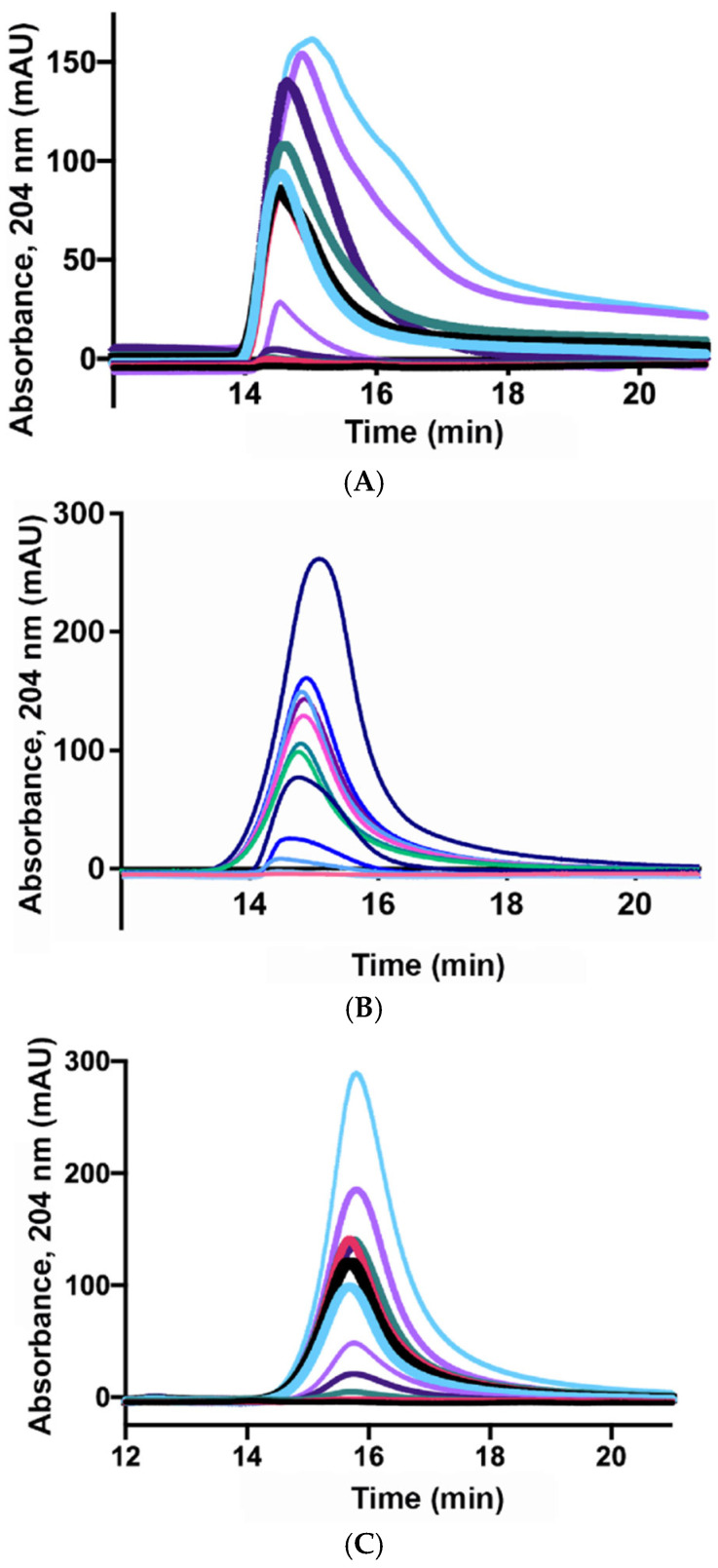
Chromatographic profiles for HMW-HA (**A**), midMW-HA (**B**) and LMW-HA (**C**) at increasing concentrations (0.0025, 0.005, 0.025, 0.05, 0.25, 0.5, 0.75, 1, 1.25, 1.5, 1.75, 2, and 2.5 mg/mL) (Reproduced from [48], under the CC BY-NC-ND 4.0 license (https://creativecommons.org/licenses/by-nc-nd/4.0/, accessed on 1 December 2025). No modifications were made).

Injected HA sample concentration. The HA concentration significantly impacts molecular weight estimations. This is especially true for HMW-HA (0.7–1.6 MDa), where errors of 11–24% have been reported even at low concentrations such as 1 g/L [102]. Previous investigations have established that highly viscous samples experience diminished separation efficiency in conventional SEC [103,104], a phenomenon referred to as viscous fingering—or, more formally, the Saffman–Taylor instability [48,105]. Such peak broadening and retention time shifts ultimately lead to producing non-linear separation behaviour. This effect is even more pronounced for HMW-HA at concentrations above 0.25 mg/mL, while in LMW-HA it becomes appreciable only above 1.0 mg/mL (Figure 5) [48]. Although viscous fingering can adversely impact separation quality, it does not preclude the use of conventional SEC for determining the molecular weight of HMW-HA; rather, it necessitates sample dilution to ensure that HA concentrations remain within the established linear range. Hence, the infinite dilution technique (described below) could be used to overcome such limitations of conventional SEC as previously proposed [47].

**Figure 6:**
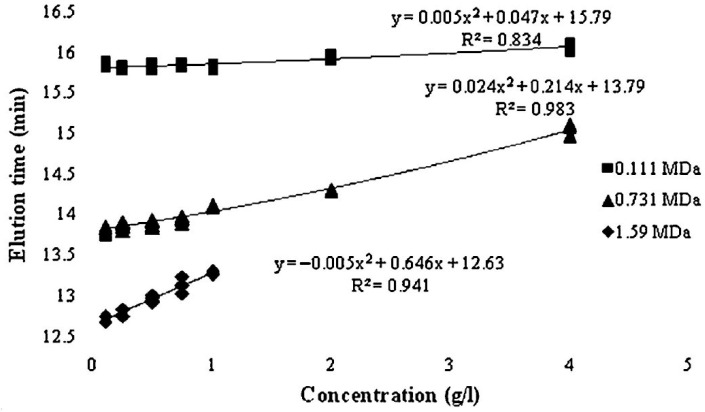
Extrapolation to infinite dilution to obtain the true elution time of HA standards [47].

The estimation of HA molecular weight by conventional SEC using a calibration curve constructed using the peak position method, which measures the elution time of the polymer at different concentrations, has been previously proposed [47]. Then, the elution time is extrapolated to infinite dilution following Lambert’s methodology [106], which allows the determination of the true elution time, effectively eliminating the influence of sample concentration on molecular weight estimation (Figure 6). By determining the true elution time through extrapolation to infinite dilution, reliable calibration curves (log(Mw) vs. elution time) can be obtained, minimizing inaccuracies caused by the use of chemically unrelated standards or suboptimal conditions. This careful calibration process laid the groundwork for developing a multivariate correlation that corrects for the concentration-dependent errors often seen in molecular weight measurements of HA. What makes this approach particularly valuable is that it significantly enhances the accuracy of conventional SEC, bringing its reliability closer to that of more advanced methods like SEC-MALLS—yet without requiring complex instrumentation or high-cost detectors.

Considering these constraints, the use of non-HA calibration standards in conventional SEC is strongly discouraged, except when used solely for comparative purposes. Accurate molecular weight determination of HA requires the development and application of HA-standards with low polydispersity and validated analytical methods tailored to the physicochemical nature of HA.

HPLC conditions affecting conventional SEC. The accuracy of SEC-HPLC methods to determine the HA molecular weight essentially depends on the column temperature and the mobile phase (flow and composition) used. To determine the best conditions to quantify HA, Suárez-Hernández et al. [92] tested the effect of four column temperatures at 30, 50, 60 and 70 °C on the repeatability and reproducibility of the method using SEC-RID. A linear dependence between HA concentration and the analytical response of the method (peak area) was found for all temperatures analyzed (correlation coefficient R^2^ > 0.99), even though the highest correlation was found at 70 °C (R^2^ = 0.9963). With increasing temperature, repeatability and reproducibility increased, which are key parameters for international acceptance of analytical methods. It has been shown that by increasing other column temperatures, there is a significant improvement in HA analysis [107]. This way, Suárez-Hernández et al. [92] showed an analytical method with a high degree of accuracy, which was validated to simultaneously estimate the concentration and molecular distribution of HA by SEC-HPLC. It was demonstrated that, at 70 °C, the repeatability and reproducibility of HA quantification increased [92]. The exclusion range of the analytical method is from 1 to 8000 polymerization degree (828 Da to 6.77 MDa). Also, for reliable hyaluronans, a high-molecular-weight distribution analysis is necessary involving SEC chromatographic columns, which must have an extremely high exclusion limit (SG-10-6000-NH_2_). Calibration of these columns should be done using hyaluronate reference material [100].

#### 2.3.2. SEC Coupled with Multi-Angle Light Scattering Detector (SEC-MALS)

Light scattering is a technique to determine the molecular weight from the angular distribution and intensity of photons interacting with macromolecules (i.e., HA). Two light scattering variants are used: MALS or dynamic light scattering (DLS). MALS is generally used for the determination of HA molecular weight (≈7 kDa up to the MDa scale), while DLS is used for particle sizing (e.g., cross-linked gels) [11]. Light scattering has the advantage of not requiring comparison to a standard for the determination of the molecular weight of the sample. The molecular weight with this technique is determined based on the intensity and angular distribution of scattered light. In particular, for MALS, current MALS detectors simultaneously measure up to 21 angles; the more angles of scattering data are used, the better the molecular weight determination. Commonly, SEC-HPLC is used in combination with a MALS detector to assess the molecular weight distribution/polydispersity of HA.

Determining the size of macromolecules with molecular weights exceeding 1 MDa by MALS requires a non-linear extrapolation of scattering intensity to zero scattering angle. This process is critically dependent on accurate measurements at low detection angles, which, in aqueous environments, are often characterized by substantial noise. Consequently, reported values of the radius of gyration (*Rg*) for HA obtained via SEC-MALS vary considerably across studies. This limitation is also reflected in the reported polydispersity indices. For low-molecular-weight HA, values in the range of 1.6–2.0 are frequently observed, consistent with near-random chain termination. In contrast, high-molecular-weight HA often yields values between 1.02 and 1.10, which, if interpreted literally, would imply an implausible degree of biosynthetic precision—on the order of accurately counting tens of thousands of residues. In reality, such results stem from insufficient resolution, whereby the molecular weight calculated for each elution time represents a heterogeneous mixture of chain lengths. When MALS is used under these conditions, the molecular weight profile across the elution peak is typically almost flat. While this would not inherently prevent the determination of an average molecular weight, the exclusion of the lowest-angle data—necessary because of high noise in aqueous samples—compromises the extrapolation to zero angle. Studies have demonstrated that omitting a single angle can produce large shifts in the fitted molecular weight. There is therefore no definitive strategy for balancing the inclusion of noisy data against the requirement for low-angle information and claims of absolute molecular weight determination for high-MW HA under such conditions should be interpreted with caution.

#### 2.3.3. Size-Exclusion Chromatography Coupled with Multiple Detectors

Size-exclusion chromatography (SEC) is usually combined with different techniques, such as light scattering (commonly MALS), refractive index (RI) and viscometry.

*Multi-angle light scattering + **refractive index detector***. To complement light scattering measurements and ensure reliable molecular weight determination, MALS systems are often coupled with an in-line differential refractometer to measure analyte concentration. Hence, following the MALS detector, an in-line RI detector is typically employed to quantify the HA concentration. This step is particularly valuable when sample quantity is limited—for example, in clinical extracts or small-scale synthetic trials—where the precise concentration is not known beforehand. Molecular weight determination relies on relating the measured scattering intensity to sample concentration through the refractive index increment (*dn*/*dc*), which is empirically established by injecting a series of HA standards of known concentration into the MALS flow cell under identical buffer conditions. To ensure reproducibility and minimize uncertainties, many researchers employ a standard buffer, such as neutral phosphate-buffered saline (0.15 M NaCl), for which *dn*/*dc* values in the range 0.153–0.167 have been well characterised. This buffer is also regarded as physiologically compatible. Additionally, a monodisperse protein such as bovine serum albumin (66 kDa) is often used to align the MALS and refractive index detector signals, calibrate the molecular weight scale, and verify the overall performance of the system [11].

In the study by Chen et al. [97], the HA molecular weight determination was performed by SEC-MALLS coupled with an in-line refractive index detector using 0.15 M NaCl as eluent and a *dn*/*dc* of 0.150 mL/g. This setup ensures absolute molar mass estimation; however, the lack of explicit alignment between MALS and RID detectors or verification with a protein standard could introduce minor systematic deviations. Although the reported dispersity indices (1.06–1.10) indicate good resolution, calibration of *dn*/*dc* under the specific pH 4.0 and ionic strength (0.2 M NaCl) conditions would enhance reproducibility and cross-study comparability.

*Multi-angle light scattering + refractive index + **viscometer detector***. In addition, most modern HA characterization systems incorporate a third, in-line viscometer detector, which provides complementary hydrodynamic information that cannot be obtained from light scattering alone. By measuring the intrinsic viscosity ([η]) of the eluting fractions, the viscometer enables the estimation of parameters such as the hydrodynamic radius (*Rh*) and the Mark–Houwink constants, offering insight into molecular conformation and flexibility in solution [93]. For HA, these measurements are particularly relevant because its extended, semi-rigid polysaccharide structure and high degree of hydration strongly influence its solution viscosity. Monitoring intrinsic viscosity across the chromatographic profile also allows detection of structural or conformational changes (e.g., chain degradation, aggregation, or branching) that may not produce large shifts in molar mass but can significantly affect rheological properties and biological function.

In the work of Hafsa et al. [98], HA extracted from rooster comb was analyzed by SEC coupled to MALS, RI, and viscometer detectors, providing a detailed physicochemical view of ultrasonic degradation. The triple-detection system enabled simultaneous determination of molecular weight, hydrodynamic radius, and intrinsic viscosity, showing a pronounced decrease in Mw (1.09–0.18 MDa) and [η] (1550–473 mL/g) after sonication. This agrees with the expected chain scission mechanism and reduced molecular expansion. Using 0.1 M LiNO_3_ and a *dn*/*dc* of 0.155 mL g^−1^ ensured detector consistency, while the overlap of MALS and viscometric profiles confirmed that ultrasonication caused linear depolymerization without affecting HA conformation. The combined MALS–RI–viscometer approach thus provides complementary hydrodynamic and conformational insights essential for linking molecular compaction and viscosity loss to functional changes in low-molecular-weight HA.

*Multi-angle light scattering + refractive index +* **UV**

In addition to viscometric coupling, SEC systems integrating MALS with RID and UV detectors provide an expanded analytical window for HA characterization. In the study by Holubova et al. [99], the combined use of MALS, RID and UV detection enabled a comprehensive evaluation of HA degradation kinetics and product homogeneity. The authors monitored the decrease in molar mass from 1.5 to 1.8 MDa to define intermediate fragments using a tri-detector setup consisting of a MALS, RID and a UV detector at 210 nm. This configuration allowed simultaneous quantification of concentration and spectral absorbance, confirming the reproducibility of papain-mediated depolymerization and the absence of chromophoric impurities. The stable baselines between RID and UV signals reflected consistent solute detection, while MALS provided accurate molar mass distribution independent of standards. Such integration of optical detectors enhances the reliability of quantitative SEC–MALS measurements, particularly when assessing purity and structural integrity in enzyme-derived HA fragments intended for biomedical use.

Diode array detector + refractive index

Beyond light-scattering configurations, simpler SEC setups combining DAD-UV detection with a RID remain valuable tools for monitoring HA purity and degradation. Although they do not provide absolute molar-mass information, the dual optical detection offers rapid, concentration-sensitive profiling and spectral confirmation of sample integrity, particularly useful for LMW-HA fractions or routine quality control analyses.

In the application note by Gough and Plankeele [108], HA was analyzed by SEC using dual detection with a UV detector at 232 nm and an RID. This configuration allowed the analysis of LMW-HA species and their comparison with reference saccharides, demonstrating reliable separation and detector response. The combination of UV and RID detection provided both concentration-sensitive and spectrally selective information, allowing differentiation between polymeric and monomeric components without resorting to a MALS detector. The stable and reproducible baselines between detectors demonstrated excellent chromatographic performance and mobile-phase compatibility (12 mM ammonium acetate, pH 6.8). Although lacking absolute molar-mass determination, the dual-detector setup offered a rapid and robust qualitative method to monitor HA degradation and confirm sample purity in low-molecular-weight fractions.

## 3. Final Remarks

When evaluating the analytical techniques used to determine the molecular weight of HA, it becomes clear that each methodology—viscometry, electrophoresis, and size-exclusion chromatography (SEC)—provides distinct yet complementary information about HA’s physicochemical behavior. Taken together, these approaches illustrate that no single technique can comprehensively describe the polymer’s molecular distribution, conformation, and structure–function relationships; instead, an integrated analytical strategy yields the most reliable and interpretable results.

Viscometry remains the most standardized and reproducible approach for average molecular weight determination and is the method recognized by major Pharmacopoeias. Through the Mark–Houwink–Sakurada relationship, intrinsic viscosity ([η]) directly reflects hydrodynamic volume and polymer flexibility, offering insight into HA conformation and degradation state. However, viscometry does not provide molecular weight distribution profiles and can be influenced by ionic strength and solvent conditions. Agarose gel electrophoresis complements this limitation by visually resolving HA fractions across a wide molecular weight range (typically 0.2–6 MDa) and enabling a qualitative assessment of polydispersity. Despite its simplicity and low cost, its accuracy depends on the use of reliable HA ladders and appropriate staining protocols, and it is not quantitative. Size-exclusion chromatography (SEC), especially in high-performance configurations (SEC-HPLC, SEC–MALS–RID–viscometry), offers quantitative characterization of molecular weight distribution and provides absolute molar mass, intrinsic viscosity, and conformational data when coupled with multi-detector systems. Nonetheless, precision at very high molecular weights can be affected by scattering noise, solvent effects, and *dn*/*dc* variability.

Interestingly, the study by Nguyen et al. [48] represents an exemplary model of integrative analysis, as it combined SEC-HPLC and agarose gel electrophoresis within a single investigation. By applying both methods to the same commercial ophthalmic products, the authors demonstrated strong concordance between chromatographic and electrophoretic profiles, while highlighting the practical advantages of SEC-HPLC for quantifying HA directly in complex, non-purified formulations. Their approach underscores the importance of using complementary analytical tools to validate molecular weight determinations and to link them to functional or biological outcomes—in this case, the effects of HA molecular form on corneal epithelial wound healing.

Such studies are particularly valuable because they bridge methodological rigor with translational relevance. The use of multiple analytical techniques enhances confidence in molecular weight data, enables inter-method calibration, and supports the establishment of reference standards for HA characterization in both research and industry. Consequently, while SEC–MALS–RID–viscometry remains the most comprehensive technique for full physicochemical profiling, the integration of electrophoresis and viscometry provides a multidimensional framework essential for standardization, regulatory compliance, and the development of HA-based biomedical formulations.

Future developments in the molecular weight characterization of hyaluronic acid (HA) are expected to focus on enhancing analytical accuracy, reducing sample preparation time, and improving the ability to resolve highly polydisperse or ultra-high-molecular-weight fractions. Emerging approaches such as multi-detector SEC configurations, advanced light-scattering technologies, microfluidic platforms, and AI-assisted data interpretation may further refine MW distribution analysis. In addition, standardized protocols and reference materials will be essential to ensure reproducibility across laboratories and to support regulatory acceptance. Continued innovation in these areas will contribute to more reliable characterization of HMW-HA, which remains a priority due to its growing relevance in biomedical and pharmaceutical applications.

## 4. Conclusions

Accurate characterization of the molecular weight of hyaluronic acid remains a critical aspect in understanding and optimizing its biological activity and therapeutic potential. The diversity of hyaluronic acid’s functional roles—ranging from structural support to signaling modulation—relies heavily on its molecular weight distribution, making robust analytical strategies indispensable. Recent advancements in electrophoresis, size-exclusion chromatography, and viscometry techniques have significantly improved the resolution, sensitivity, and reproducibility of the hyaluronic acid’s molecular weight determination. The integration of high-resolution columns, multi-detector systems, refined empirical formulas, and enhanced gel matrices has enabled a more comprehensive and reliable analysis of hyaluronic acid, even in highly heterogeneous or polydisperse samples. These innovations support a more holistic and multidimensional approach to hyaluronic acid’s molecular weight characterization, bridging gaps between analytical precision and biological relevance. Future trends should emphasize the further refinement and cross-integration of analytical techniques, which will be critical for the standardization of hyaluronic acid-based formulations and the development of novel therapeutic strategies. The intersection of advanced instrumentation, robust quantitative analysis, and interdisciplinary methodologies is expected to drive a more sophisticated, application-specific characterization of hyaluronic acid.

## Figures and Tables

**Table 1:** Comparison of analytical techniques used to determine the molecular weight (MW) of hyaluronic acid (HA).

Technique	Advantages	Limitations	Comments
Viscometry	Officially recognised in pharmacopoeias Simple and inexpensive Reflects rheological properties Used in industrial QC	Only provides average MW Requires large sample amounts (~1 g) Sensitive to temperature and ionic strength No MW distribution	Widely accepted for regulatory purposes, but not suitable for polydisperse or complex HA samples.
Gel electrophoresis (agarose/PAGE)	Inexpensive and widely available Provides full MW distribution and polydispersity Tolerates moderate sample impurities Requires small sample amounts (µg)	Low sensitivity for <4.4 kDa HA Non-specific staining may interfere Requires reliable MW standards Needs optimization by gel type	One of the most practical, accessible, and informative methods for MW profiling is when well-optimized.
SEC-HPLC (RI detector)	Compatible with standard HPLC Produces MW distribution chromatogram Easy to operate and low cost after setup Good for product comparisons	Highly sensitive to calibration standard used: pullulan and PEO can overestimate by 2–10× Concentration-dependent errors up to 58% Poor resolution for very high MW (>2 MDa)	Only reliable if using HA-based calibration and correcting for concentration effects (see [47]).
SEC-MALLS	Absolute MW measurement (no calibration needed) Wide MW range (7 kDa to several MDa) High accuracy for average MW	Expensive instrumentation Requires purified samples Resolution limited by the SEC step Low-angle light scattering subject to noise	Considered the “gold standard” for MW, though less precise for distribution unless combined with high-resolution separation.
GEMMA (Gas-phase electrophoretic mobility)	Extremely sensitive for low MW (30–2400 kDa) Requires picogram-level sample Fast (5–10 min) Single calibration needed	Assumes spherical conformation (can bias MW estimates) Underestimates > 70 kDa HA Limited distribution detail Requires pure HA	Ideal for analyzing HA in low-concentration biological fluids; best for comparative rather than absolute analysis.
MALDI-TOF MS	High sensitivity Capable of detecting oligomers Rapid spectral acquisition	Only effective for small HA (<10 kDa) Requires pure samples Not quantitative for polydisperse mixtures High instrument cost and expertise needed	Effective for profiling small HA fragments; not applicable for large or complex HA distributions.
FFF (Field Flow Fractionation)	Excellent for ultra-high MW HA (>1–2 MDa) Reduces shear degradation Can be coupled with MALLS	High cost Limited availability Lower throughput than SEC	Superior to SEC for very large HA polymers, especially when high resolution and gentle separation are required.
Nanopore analysis (solid-state)	Single-molecule sensitivity Works with small samples (~10 ng) Broad MW range (up to 20 MDa) Simultaneous MW distribution and quantification possible	Expensive and technically complex Requires highly purified HA Data analysis is computationally demanding Risk of pore clogging with very large HA	Emerging technique with great potential for full-spectrum HA characterization. Not yet routine but rapidly advancing.

**Table 2:** Published *k* and *α* parameters in the Mark–Houwink equation for the determination of the intrinsic viscosity ([*η*], in dL/g) of hyaluronic acid (HA).

HA Molecular Weight (Da)	*k* (dL/g)	*α*	Solvent *	Reference
0.077–1.7 × 10^6^	0.36 × 10^−3^	0.78	0.1 M NaCl	[69]
>0.1 × 10^6^	0.228 × 10^−3^ 0.318 × 10^−3^	0.816 0.777	0.2 M NaCl 0.5 M NaCl	[70]
0.31–1.5 × 10^6^	0.57 × 10^−3^	0.76	0.5 M NaCl	[71]
0.25–1.63 × 10^6^	0.39 × 10^−3^	0.77	0.2 M NaCl	[72]
>2.4 × 10^6^	0.16 × 10^−3^	0.841	0.1 M NaCl	[52]
0.1–1.0 × 10^6^ >1 × 10^6^	0.346 × 10^−3^ 0.397 × 10^−3^	0.779	0.15 M NaCl	[58]
0.4–2.66 × 10^6^	0.199 × 10^−3^	0.829	0.2 M NaCl	[73]
0.42–1.38 × 10^6^	0.278 × 10^−3^	0.78	0.1 M NaNO_3_	[51]
-	0.29 × 10^−3^	0.80	-	[74]
-	0.57 × 10^−3^	0.75	0.15 M NaCl	[75]
0.5–1.49 × 10^6^ 1.5–3.9 × 10^6^	0.36 × 10^−3^ 0.228 × 10^−3^	0.78 0.816	0.2 M NaCl	[76]

* aqueous solution.

**Table 3:** Agarose gel electrophoresis methods for the determination of the molecular weight of hyaluronic acid (HA).

Agarose Gel (%)	Buffer	Pre-Run (Power/Time)	Run (Power/Time)	Staining Conditions	Destaining Conditions	HA Standards	Ref.
0.5	TAE *	-	20 V/30 min 40 V/210 min	Stains-All	-	HA standards (0.2–6 MDa)	[46]
1.2	TBE ** (pH 8.2)	-	100 V/300 min	0.05% Stains-All (in 50% ethanol)	in water (2 h) + light	-	[85]
0.5	-	-	100 V/60 min	0.1 mg/mL Stains-(in 30% ethanol)	light	-	[86]
0.5	TAE (pH 8)	-	105 V/55 min	0.003% Stains-All (in 30% ethanol)	in water + light	Select-HA HiLadder™ (HA ≥ 2 MDa)	[82]
0.5	TAE (pH 7.9)	30 V/30 min	50 V/360 min	0.005% Stains-All (in 50% ethanol)	in water (4 h) + light	MEGA/High Ladder, HMW HA (1.63 MDa), digested AF controls	[89]
0.5	TAE	-	50 V/480 min	0.005% Stains-All (in 50% ethanol)	in water (48 h) + light	-	[88]
1.0	TBE (1×)	0.08 V/360 min	0.1 V/60 min	5% Stains-All	light	HA MW standard	[84]
0.5	TAE (pH 8)		105 V/60 min	6.25 µg/mL Stains-All (in 30% ethanol)	in water (24 h) + light	Select-HA HiLadder™, HA ≥ 2 MDa	[83]

* TAE, Tris-Acetate-EDTA; ** TBE, Tris-Borate-EDTA.

**Table 4:** Size-Exclusion High-Pressure Liquid Chromatography (SEC) (with different detectors and their combinations) for the determination of the molecular weight of hyaluronic acid (HA).

Molecular Weight Reported (MDa)	Microorganism	HA Sample Concentration (g/L)	Extraction Buffer	HPLC Conditions	Calibration Standard Used (Concentration)	Column	Detector	Ref.
SEC-RID								
2–5 MDa	*Streptococcus equi* KFCC 10830	-	-	-	Pullulan (conc. NA)	TSK 5000 PW (up to 1 MDa; Toyo Soda) + TSK 6000 PW (up to 8 MDa; Toyo Soda) *Material:* hydroxylated methacrylic polymer functionalized with quaternary amine strong anion exchange groups	RID	[94]
5.2–15.4 MDa	*S. equi* ATCC 39920	-	-	0.1 M NaNO_3_ 0.6 mL/min; 37 °C	Pullulan (conc. NA)	Ultrahydrogel Linear (up to 7 MDa; Waters) *Material:* Crosslinked hydroxylated polymer containing residual carboxyl functionality	RID	[95]
0.111–2.67 MDa (standards); 0.140–1.644 MDa corrected (“true MW-SEC”)	*Lactococcus lactis*	0.1–10	Ethanol-precipitated HA, redissolved in mobile phase	0.2 M NaNO_3_; 0.6 mL/min; ambient temperature; 20 μL injection	Pullulan (0.112–0.788 MDa; 0.1–1 g/L) PEO (0.6–8 MDa; 0.1–1 g/L) HA (0.111–2.67 MDa; 0.1–6 g/L)	Phenomenex GFC-P 6000 (300 × 7.8 mm) + Phenomenex GFC-P guard (30 × 7.8 mm) *Material:* hydroxylated methacrylate polymer (hydrophilic gel matrix) designed for aqueous GPC/SEC separations; neutral, non-ionic resin suitable for polysaccharides and glycosaminoglycan	RID	[47]
**SEC-UV**								
0.1 → 2.5 MDa (OTC products); standards 0.004–2.67 MDa	-	0.14–4.0 mg/mL (products); 0.0025–2.5 mg/mL (standards)	-	0.1 M sodium phosphate + 20 μM EDTA, pH 6.8; 0.300 mL/min; 35 °C; 25 μL injection	HA standards 4–8 kDa to 2670 kDa, 2.5 μg/mL–2.5 mg/mL	2 × AdvancedBio SEC 300 Å (4.6 × 300 mm, silica, Agilent) + 1 × Zorbax GF-250 (4.6 × 250 mm, silica, Agilent), in tandem	UV 204 nm (also 280 nm)	[48]
-		2.0 mg/mL (formulations); 0.32–0.48 mg/mL (standards)	0.05 M KH_2_PO_4_	0.05 M KH_2_PO_4_, pH 7.0 1.0 mL/min; 25 °C; 10 μL injection	Sodium hyaluronate reference standards (0.32–0.48 mg/mL; BPCL, London)	BioSep SEC S2000 (300 × 7.8 mm, silica-based diol phase, Phenomenex)	UV (205 nm)	[96]
**SEC with combined detectors**								
0.1–1 MDa	-	-	0.15 M NaCl	0.15 M NaCl; 0.5 mL/min	-	-	MALS+RID	[97]
0.5–1.5 MDa	-	0.5 g/L	NaOAc 5%	0.1 M LiNO_3_; 0.5 mL/min; 25 °C 200 µL of 0.5 mg/mL HA solution	-	Shodex OHpak SB-804 HQ and Shodex OHpak SB-806 HQ (Showa Denko K.K., Tokyo, Japan) 8 mm I.D. × 300 mm length (each) Two columns connected in series	MALS+VD+RID	[98]
0.01–1.2 MDa	*Streptococcus equi*	0.75 g/L	0.1 M phosphate buffer + 0.1 M NaCl + 1.5 mM saccharic acid-1,4-lactone (pH 7.0)	50 mM sodium phosphate+ 3.1 mM sodium azide; 0.8 mL/min; 100 µL injection	-	-	MALS+RID+UV	[99]

“NA” indicates that the concentration of the calibration standard was *not available* or *not specified* in the original source.

## Data Availability

No new data were created or analyzed in this study.
